# Causal relationship between Parkinson’s disease and gastric cancer: a Mendelian randomization study

**DOI:** 10.1186/s12883-025-04184-7

**Published:** 2025-04-16

**Authors:** Jiayu Liu, Jiafeng Liu, Lanjun Li, Wenju Li, Ziyang Jiang, Gang Yin, Yunling Zhang, Yuan Sun

**Affiliations:** 1 Department of Neurology, Rizhao Central Hospital, Rizhao, 276800 Shandong China; 2 Department of Neurology, Jingzhou Hospital Affiliated to Yangtze University, Jingzhou, 434400 Hubei China; 3 Department of Neurology, Xuzhou Central Hospital, Xuzhou, 221000 Jiangsu China

**Keywords:** Parkinson’s disease, Gastric cancer, Mendelian randomization, P2X6

## Abstract

**Background:**

Age-related diseases, including Parkinson’s disease and gastric cancer, substantially affect the global aging population. Previous observational research has suggested a potential association between Parkinson’s disease and gastric cancer; however, findings regarding this aspect have been inconsistent. In the present study, we used data from genome-wide association studies to infer a causal relationship between Parkinson’s disease and gastric cancer based on genetic variations.

**Methods:**

We used the two-sample Mendelian randomization method to analyze data from the genome-wide association study catalog, including 482,730 and 476,116 patients with Parkinson’s disease and gastric cancer, respectively. Inverse-variance weighting was used as the primary Mendelian randomization analysis. We conducted sensitivity analyses to evaluate heterogeneity and pleiotropy, followed by two-step Mendelian randomization to ascertain the latent mediator of the relationship between Parkinson’s disease and gastric cancer.

**Results:**

Our results suggested a causal negative relationship between Parkinson’s disease and gastric cancer. Heterogeneity and pleiotropy analyses confirmed the robustness of the inverse-variance weighting results. Furthermore, P2X6 was identified as a key factor mediating this negative causal relationship.

**Conclusions:**

Patients with Parkinson’s disease may have a lower risk of developing gastric cancer, with P2X6 serving as a significant mediating variable. These novel insights can aid the development of potential therapeutic targets for patients with Parkinson’s disease or gastric cancer.

## Background

Parkinson’s disease (PD) is an age-related neurodegenerative disease [1, 2]. Its global age-standardized prevalence rate increased by 21.7% from 1990 to 2016 [1]; with the increase in the global aging population [3], its incidence rate is expected to increase substantially over the next 20 years, posing a huge burden on families and societies [1]. According to GLOBOCAN 2020, the incidence and mortality rates of gastric cancer have declined globally [4]. Although PD and gastric cancer are both age-related diseases [1, 5], their causal relationship remains unclear. Previous studies using gene expression profiles and bioinformatic analyses have suggested no potential relationship between PD and gastric cancer [6]. However, other studies have concluded that patients with PD may have a high risk of developing gastric cancer [7]. These inconsistent results may be attributed to different confounding factors.

Mendelian randomization (MR) is a method used to evaluate causal relationships by analyzing genetic variations associated with biological intermediates of interest [8]. In the present study, we aimed to reveal the causal relationship between PD and gastric cancer using two-sample MR and identify the intermediate variables between the two diseases using two-step MR.

## Methods

### Two-sample MR analysis

**Fig. 1:**
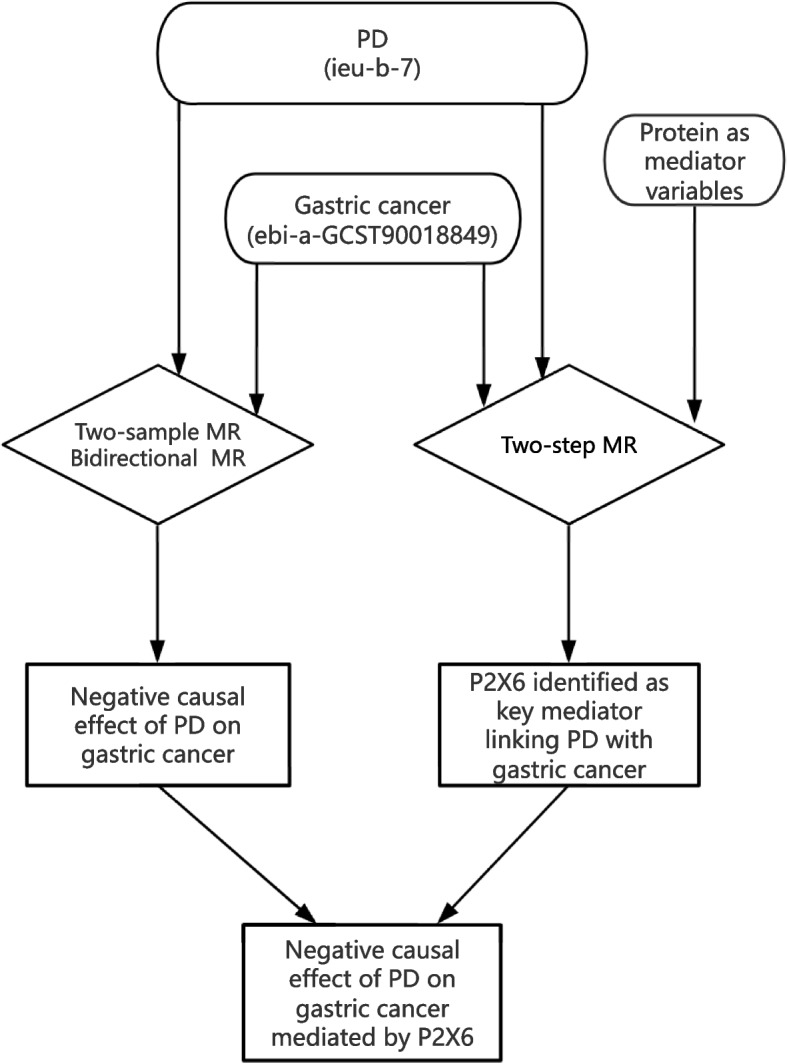
Summary of the protocol used in the present study. PD and gastric cancer were analyzed through two-sample MR. PD exhibited a negative causal effect on gastric cancer. P2X6 was identified as an important mediator of the effects of PD on gastric cancer. MR: Mendelian randomization; PD: Parkinson’s disease

Two-sample MR was used to detect the potential relationship between PD and gastric cancer. In the first part, PD was considered the exposure of interest, with gastric cancer as the outcome. Conversely, in the second part, gastric cancer was considered the exposure of interest, with PD as the outcome (Fig. 1). Single-nucleotide polymorphisms (SNPs) served as instrumental variables (IVs).

The two-sample MR method was based on the following assumptions: (I) IVs are strongly associated with exposure (PD/gastric cancer); (II) IVs affect the outcome (gastric cancer/PD) solely through their influence on the exposure; and (III) IVs are independent of confounding factors.

Summary-level data were obtained from the genome-wide association study (GWAS) catalog [9, 10], and both groups included only European individuals. The GWAS data for PD (ieu-b-7) [9] included data corresponding to 33,674 patients with PD and 449,056 controls, with 17,891,936 SNPs, which were used as summary association statistics for exposure (Table 1). The GWAS data for gastric cancer (ebi-a-GCST90018849) [10] included data corresponding to 1,029 patients with gastric cancer and 475,087 controls, with 24,188,662 SNPs, which were used as the summary association statistics for the outcome (Table 1).

**Table 1:** GWAS data for Parkinson’s disease and gastric cancer

Disease	Database	SNP GWAS	Cases	Controls	Year
Parkinson’s disease	ieu-b-7	17,891,936	33,674	449,056	2019
Gastric cancer	ebi-a-GCST90018849	24,188,662	1,029	475,087	2021

*GWAS* Genome-wide association study, *SNP* Single-nucleotide polymorphism

In our MR approach, we used genetic variations associated with exposure as IVs to explore the potential causal relationship between exposure and outcome. The primary MR estimation method used was the multiplicative random effects inverse-variance weighting (IVW) method, supplemented by the MR-Egger, weighted median, simple mode, and weighted mode methods. A *P* value < 0.05, generated by at least one of the five MR methods, was considered the significance threshold for the causal effects of PD on gastric cancer. Genetic variations associated with the risk of PD were identified using a significance threshold of *P* < 5 × 10^−8^ (a standard threshold in GWAS for minimizing false positives and ensuring robust genetic associations) within a range of 10,000 kb, characterized by low linkage disequilibrium (r^2^ < 0.001) and strong IV strength (F statistic > 10).

### Two-step MR analysis

A two-step MR analysis was performed to identify the candidate proteins (variables) mediating the causal relationship between PD and gastric cancer (Fig. 1). The GWAS data for proteins were obtained from the GWAS catalog (prot-a-2160). In the two-step MR analysis, the following equation was used as the direct effect of exposure on the outcome: β0 − β1 × β2, where β0 represents the total causal effect of the exposure on the outcome, β1 represents the causal effect of exposure on the mediator, β2 represents the causal effect of the mediator on the outcome, and β1 × β2 indicates the mediating effects of exposure on the outcome.

Directional pleiotropy was evaluated based on the intercept obtained from the MR-Egger analysis. All analyses were performed using the TwoSampleMR package (version 0.5.6) in R software (version 4.2.1; R Foundation for Statistical Computing, Vienna, Austria).

## Results

### MR analysis revealed a negative causal effect of PD on gastric cancer

**Fig. 2:**
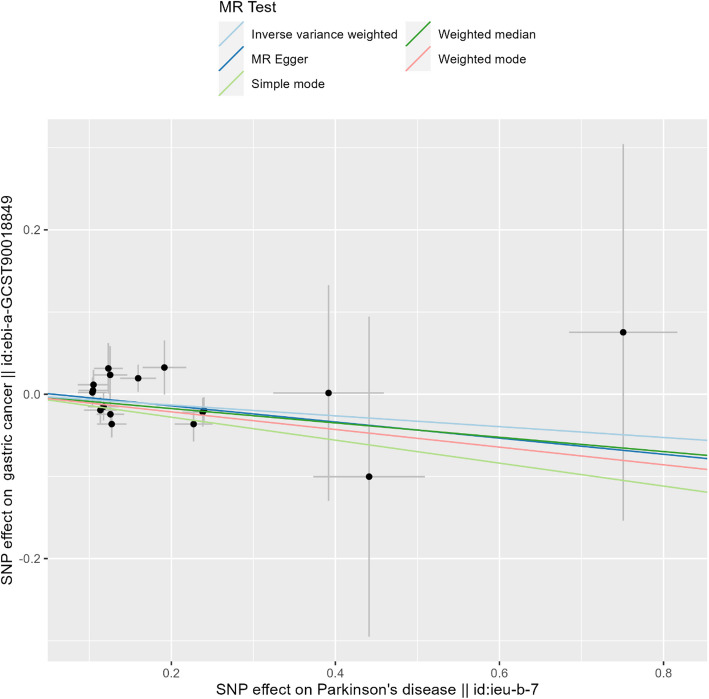
Causal effects of PD on gastric cancer. MR: Mendelian randomization; PD: Parkinson’s disease

**Fig. 3:**
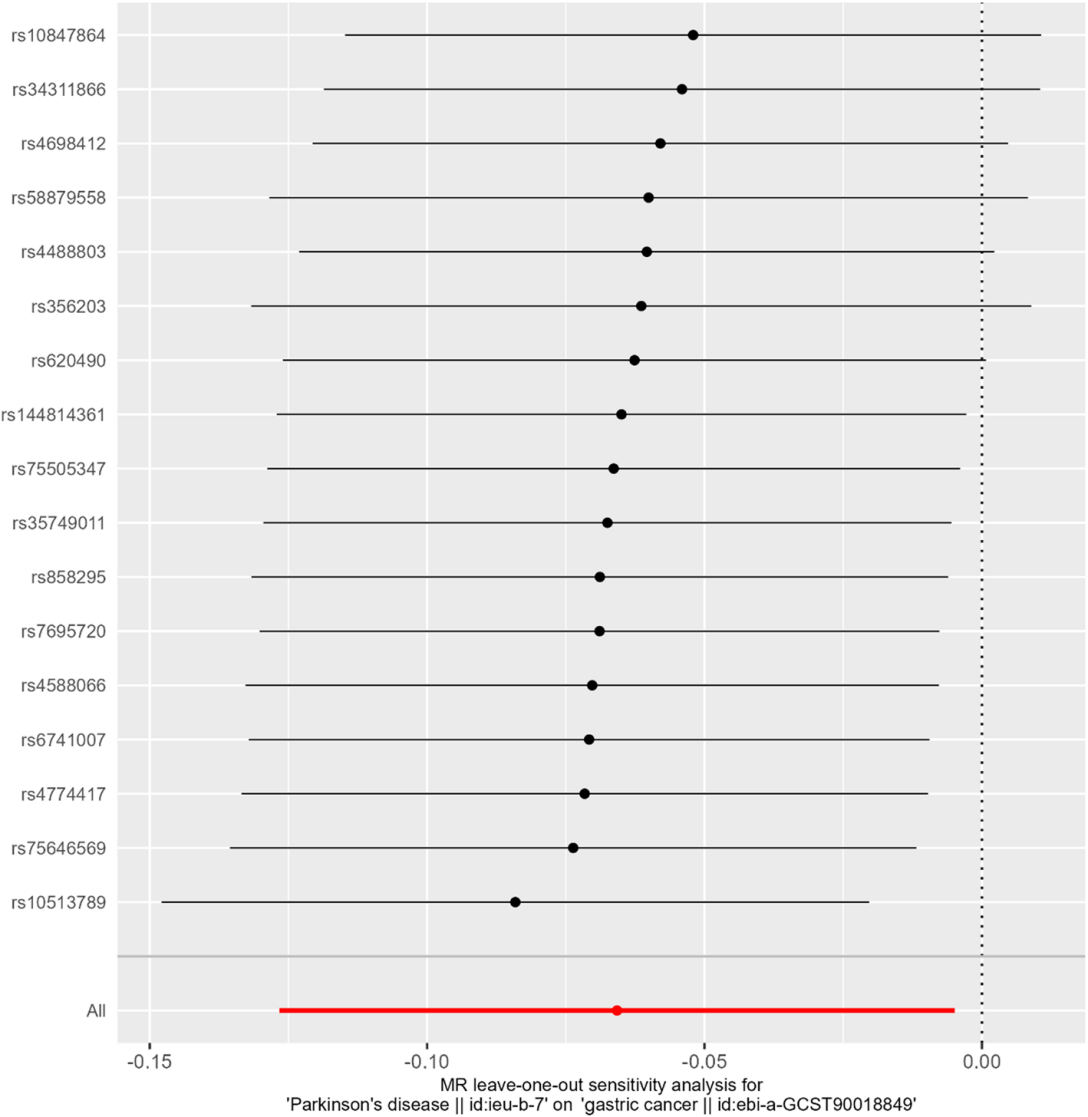
Results of the leave-one-out analysis. The X-axis indicates the analysis results of the random effects. The Y-axis represents the reference SNP cluster identification

We identified a negative causal relationship between PD and gastric cancer. In the two-sample MR analysis, using PD as the exposure, gastric cancer as the outcome, and SNPs as the IVs, our results showed that PD could reduce the risk of gastric cancer (*P*_IVW_ = 0.034, *P*_MR-Egger_ = 0.307, *P*_weighted median_ = 0.050, *P*_simple mode_ = 0.081, and *P*_weighted mode_ = 0.033; Table 2; Fig. 2). In our comprehensive MR analyses, the results of the Cochran’s Q test suggested the absence of heterogeneity (Cochran’s Q_MR-Egger_ = 15.55 and *P* = 0.41; Table 2). The leave-one-out sensitivity analysis revealed that no single SNP significantly violated the overall effect of PD on gastric cancer (all *P* values ≤ 0), confirming the absence of significant heterogeneity (Fig. 3). Furthermore, horizontal pleiotropy analysis showed little evidence of pleiotropy in this association (*P* = 0.715; Table 2).

**Table 2:** Two-sample MR results for Parkinson’s disease as the exposure and gastric cancer as the outcome

	**Method**	**Nsnp**	**β**	**SE**	***P* value**
**Results**	MR-Egger	17	− 0.09820	0.092921	0.307307
	Weighted median	17	− 0.08722	0.044517	0.050085
	Inverse-variance weighting	17	− 0.06577	0.031052	0.034177
	Simple mode	17	− 0.13972	0.075184	0.081612
	Weighted mode	17	− 0.10731	0.046047	0.033197
	**Method**	**Q**	**Q_df**	**Q_pval**	
**Heterogeneity test**	MR-Egger	15.55314	15	0.412355	
	Inverse-variance weighting	15.69603	16	0.47437	
	**egger_intercept**	**SE**	**pval**		
**Test for directional horizontal pleiotropy**	0.005488	0.014782	0.71566		

*MR* Mendelian randomization, *Nsnp* Number of single nucleotide polymorphisms, *B* Beta, *SE* Standard error, *Q* Cochran’s Q statistic, *Q_df* Q degrees of freedom, *Q_pval* Q *P* value

No potential causal effect of gastric cancer on the risk of PD was observed using gastric cancer as the exposure, PD as the outcome, and SNPs as the IVs (*P*_IVW_ = 0.989, *P*_MR-Egger_ = 0.794, *P*_weighted median_ = 0.867, *P*_simple mode_ = 0.894, and *P*_weighted mode_ = 0.779; Table 3).

**Table 3:** Two-sample MR results for gastric cancer as the exposure and Parkinson’s disease as the outcome

	**Method**	**Nsnp**	**Β**	**SE**	***P* value**
**Results**	MR-Egger	5	-0.09001	0.31633	0.79451
	Weighted median	5	0.01495	0.08951	0.86740
	IVW	5	0.00106	0.07941	0.98938
	Simple mode	5	0.01564	0.11036	0.89418
	Weighted mode	5	0.02944	0.09816	0.77913

*IVW* Inverse-variance weighting, *MR* Mendelian randomization, *Nsnp* Number of single nucleotide polymorphisms, *B* Beta, *SE* Standard error

### Identification of mediator variables between PD and gastric cancer

To explore the potential mechanism underlying the causal effects of PD on gastric cancer, we performed a two-step MR analysis. Using the IVW method, we found that P2X6 was an important mediating variable in the association between PD and gastric cancer (Table 4). In particular, we determined that PD exerted a positive causal effect on P2X6 (*P*_IVW_ < 0.01; β > 0), which, in turn, exhibited a negative causal effect on gastric cancer (*P*_IVW_ < 0.05; β < 0; Table 4).

**Table 4:** Two-step MR results for P2X6 as a mediator variable for PD and gastric cancer

	**Nsnp**	**β**	**SE**	***P* value**
PD as the exposure and P2X6 as the outcome	17	0.100569	0.047194	0.033094
P2X6 as the exposure and gastric cancer as the outcome	1	− 0.191464	0.096028	0.046171
	**Mediating effect**	**Direct effect**		
PD as the exposure, gastric cancer as the outcome, and P2X6 as the mediator	− 0.0193	− 0.04647		

*MR* Mendelian randomization, *PD* Parkinson’s disease, *Nsnp* Number of single nucleotide polymorphisms, *B* Beta, *SE* Standard error

## Discussion

In the present MR-based study, we used extensive data from the GWAS catalog to provide novel insights into the potential causal relationship between PD and gastric cancer. Although previous observational studies have hypothesized a possible link between PD and gastric cancer [6, 7], the results were often confounded by various uncontrollable factors and reverse causality issues. In this study, we used the MR methodology using genetic variants as IVs, overcoming these limitations and revealing the causal relationship between the two diseases.

We observed a negative correlation between PD and the risk of gastric cancer, suggesting that individuals with PD may have a reduced risk of developing gastric cancer than the general population. Our finding was inconsistent with those of previous observational studies, which suggested that patients with PD have a higher risk of developing gastric cancer [7]. However, our MR analysis offers a different perspective, suggesting that this relationship may not be directly causal but rather mediated by shared genetic backgrounds or other biological mechanisms.

The identification of P2X6 as a key mediating variable provides clues to understanding the potential biological connections between PD and gastric cancer. P2X6 is a purinergic receptor involved in inflammatory processes [11, 12], suggesting that inflammatory pathways may contribute to a reduced risk of gastric cancer being associated with PD. While its role in PD and gastric cancer is not fully understood, previous studies have suggested that purinergic signaling may play a role in neurodegeneration and cancer progression. Moreover, this discovery highlights the importance of conducting further research regarding the genetic and molecular mechanisms underlying the relationship between PD and various types of cancer.

Our study has various strengths. First, using an MR design, we reduced the impact of confounding factors that are typically encountered in traditional observational studies. Second, our analysis was conducted using a large sample size, which increased the statistical power of our findings. Finally, our results were validated using various MR methods, including rigorous assessments of heterogeneity and pleiotropy, thereby improving the reliability of our conclusions.

Despite these advantages, our study also had certain limitations. MR methods are beneficial for causal inference, but they rely on the selection of IVs and the correlation between genetic variation and phenotypes. Although we identified P2X6 as a potentially important intermediary factor, further functional studies are needed to validate its role in linking PD to gastric cancer. P2X6 may also act as a confounder rather than a true mediator, as its expression may be influenced by other genetic or environmental factors. Furthermore, our mediation analysis of P2X6 was based on a limited number of SNPs, which may reduce the statistical power and increase the risk of horizontal pleiotropy. Future studies should use multiple independent SNPs to validate these findings. Finally, the GWAS data used in this study were obtained from European populations, which limits the generalizability of our findings to other ethnic groups. Future studies should evaluate diverse populations to validate the observed associations and ensure broader applicability.

The findings of our MR study provide novel evidence for understanding the complex relationship between PD and gastric cancer, suggesting potential directions for the prevention and treatment of, and development of therapeutic targets for, these conditions.

## Conclusions

We showed that PD may be associated with a reduced risk of developing gastric cancer; P2X6 may be an important intermediate variable associated with PD and gastric cancer.

## Data Availability

All data generated or analyzed during this study are included in this published article.

## Abbreviations

GWAS: Genome-wide association study
IV: Instrumental variables
IVW: Inverse-variance weighting
MR: Mendelian randomization
PD: Parkinson’s disease
SNP: Single-nucleotide polymorphism
